# Conservative Management of Azygous Vein Rupture in Blunt Thoracic Trauma

**DOI:** 10.1155/2012/147614

**Published:** 2012-11-29

**Authors:** Cian McDermott, Gabrielle O'Connor, Eilish McGovern, Geraldine McMahon

**Affiliations:** ^1^Emergency Department, St James's Hospital, Dublin 8, Ireland; ^2^Cardiothoracic Surgery Department, St James's Hospital, Dublin 8, Ireland

## Abstract

We report a case of successful conservative management of acute traumatic rupture of the azygous vein. A 48-year-old male was involved in a motor vehicle collision. Primary survey revealed acute right intrathoracic haemorrhage. He remained haemodynamically stable with rapid infusion of warmed crystalloid solution and blood. Computed tomographic imaging showed a contained haematoma of the azygous vein. The patient was managed conservatively in the intensive care. Azygous vein laceration resulting from blunt thoracic trauma is a rare condition that carries a universally poor prognosis unless the appropriate treatment is instituted. Clinical features include acute hypovolaemic shock, widened mediastinum on chest radiograph, and a right-sided haemothorax. Haemodynamic collapse necessitates immediate resuscitative thoracotomy. Interest in this injury stems from the severity of the clinical condition, difficulty in diagnosis, the onset of a rapidly deteriorating clinical course all of which can be promptly reversed by timely and appropriate treatment. Although it is a rare cause of intramediastinal haemorrhage, it is proposed that a ruptured azygous vein should be considered in every trauma case causing a right-sided haemothorax or widened mediastinum. All cases described in the literature to date involved operative management. We present a case of successful conservative management of this condition.

## 1. Introduction

A 48-year-old male was the restrained driver of a motor vehicle involved in a head-on collision with a wall at high speed. Primary survey in the Emergency Department (ED) revealed reduced breath sounds over the right hemithorax and subcutaneous emphysema of the right chest wall. The initial blood pressure measured 100/60 mmHg, and he had a pulse rate of 84 beats/minute.

**Figure 1:**
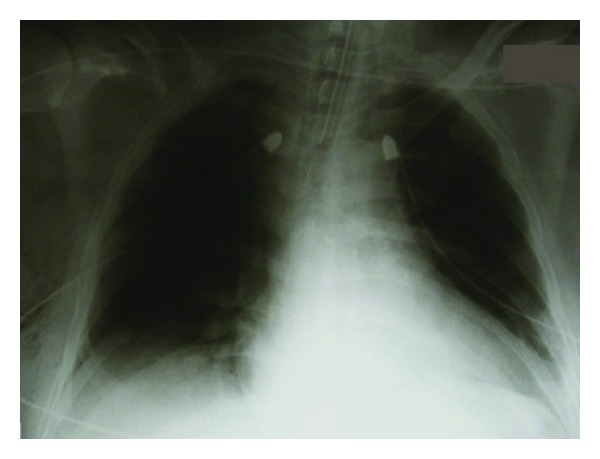
Portable chest radiograph showing a right-sided haemothorax and a widened mediastinum (bilateral chest drains inserted).

Chest radiography revealed a widened mediastinum, bilateral pulmonary contusions, and multiple rib fractures (Figure 1). The patient was resuscitated with intravenous crystalloid solution and blood to maintain haemodynamic stability. A 32-Fr thoracostomy tube was inserted in the right pleural cavity with an output of 250 mL of blood with minimal ongoing blood loss.

**Figure 2:**
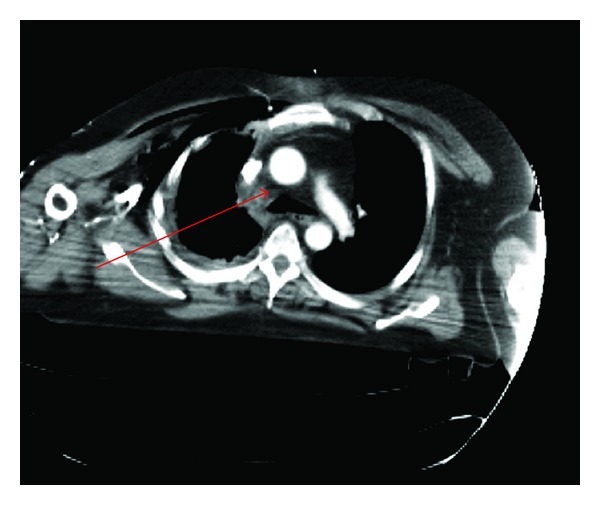
CT Thorax with a right-sided paratracheal haematoma at the level of the azygous vein (see arrow).

Whole body-computed tomographic (CT) imaging demonstrated a right-sided haemopneumothorax with extensive pulmonary contusions and multiple ipsilateral rib fractures. There was a mediastinal haematoma present in the right paratracheal area at the position of the azygous vein (Figure 2). Imaging of the head, abdomen, and pelvis were negative for further injury. Following orotracheal intubation, the patient was transferred to the intensive care unit for close observation. He was successfully stabilised with crystalloid and blood infusion, and he did not require exploratory thoracic surgery.

## 2. Discussion

Blunt thoracic trauma accounts for 25% of trauma-related mortality. Injury to the heart and great vessels of the thorax as a result of abrupt deceleration are recognised causes of immediate death [1]. Although an uncommon complication of blunt thoracic trauma, rupture of the azygous vein is associated with potentially life-threatening haemodynamic shock in 85% of cases [2]. The clinical pattern may be indistinguishable from injury to other great vessels. 

The initial clinical presentation of a ruptured azygous vein is that of shock with acute hypovolemic loss into the right thoracic cavity. A right-sided haemothorax is the most common finding on a primary survey chest radiograph. The presence of a widened mediastinum and/or a haemothorax is of diagnostic significance and is usually attributed to disruption of the aorta or its major branches. However, these findings may also occur as a result of azygous vein disruption. Diagnostic computed tomographic scans of the thorax to determine the cause of mediastinal widening is indicated, if the patient is haemodynamically stable.

**Figure 3:**
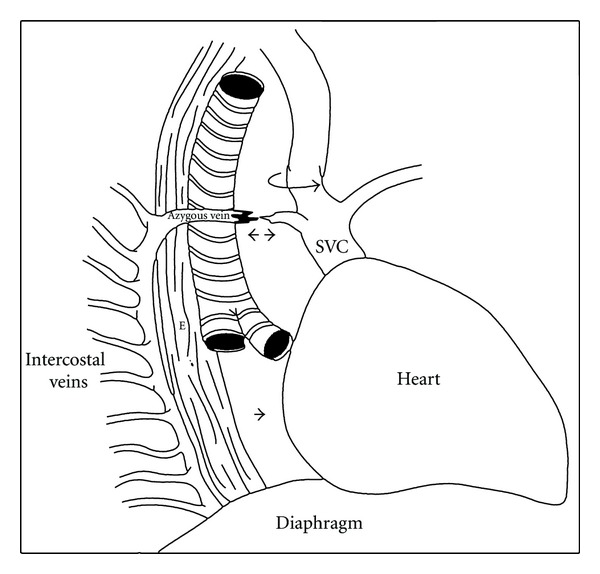
Schematic diagram illustrating the anterior and posterior attachments of the azygous vein (reproduced with permission from Annals of Thoracic Surgery).

Azygous vein injury in blunt thoracic trauma is most often a result of sudden decelerating forces such as those involved in motor vehicle accidents or falls from a height [3–5]. The azygous vein is most commonly torn at its arch, just proximal to the junction with the superior vena cava [4] (Figure 3). It is postulated that several different aetiologic mechanisms of injury may be involved. Rupture of the azygous vein may result from abrupt deceleration applied to the mobile azygous arch which may initiate shearing forces within the thorax [6, 7]. Also, an associated anterior dislocated fracture of the upper thoracic vertebrae in the proximity of the azygous vein may contribute to venous disruption [2, 4, 8]. This may have been the mechanism of injury in our patient who had also sustained a stable thoracic vertebral body fracture.

The management of a patient with a traumatic injury to the azygous vein involves rapid evaluation and resuscitation in ED in accordance with advanced trauma life support principles [1]. When suggestive clinical features or plain film abnormalities are detected in the haemodynamically unstable patient, then immediate surgical thoracotomy may prove lifesaving [2–7]. Right-sided tube thoracostomy is generally indicated for the management of a right haemothorax [2–4, 7]. An isolated contained tear of the azygous vein may respond to conservative management due to low venous pressure and intact mediastinal pleura. For the patient who is haemodynamically stable, aortography, transoesophageal echocardiography, and CT imaging of the thorax are the imaging modalities of choice to evaluate the associated aortic injuries [9].

Wall et al. described a series of 22 penetrating injuries to the azygous venous system over a 40-year period; all of these cases were managed by exploratory thoracotomy [10]. Nguyen and Drac presented a literature review of all previously reported blunt traumatic azygous vein injuries [11, 12]. Similarly, in all of these cases, operative intervention has been required to control intramediastinal haemorrhage. Our patient is unique because he did not require a thoracotomy. Thus, it represents the first recorded case in the literature outlining a successful conservative approach to the management of acute traumatic azygous vein rupture.

## 3. Conclusion

A high index of clinical suspicion is crucial for the timely detection of an azygous vein lesion. The injury may remain occult and may have potentially catastrophic consequences if the diagnosis is delayed or missed. As with all traumatic injury, the emergency physician is important in suspecting the initial diagnosis and initiating appropriate life-saving treatment. Thus, awareness of this rare condition with early diagnosis and active aggressive management may improve eventual patient outcome.
